# Corneal injury repair and the potential involvement of ZEB1

**DOI:** 10.1186/s40662-024-00387-0

**Published:** 2024-06-01

**Authors:** Lin Jin, Lijun Zhang, Chunxiao Yan, Mengxin Liu, Douglas C. Dean, Yongqing Liu

**Affiliations:** 1 Department of Ophthalmology, The Third People’s Hospital of Dalian, Dalian Medical University, Dalian, 116033 China; 2 James Brown Cancer Center, University of Louisville School of Medicine, Louisville, KY 40202 USA; 3 Department of Medicine, University of Louisville School of Medicine, Louisville, KY 40202 USA

**Keywords:** ZEB1, EMT, Corneal damage, Inflammation, Neovascularization, Scarring

## Abstract

The cornea, consisting of three cellular and two non-cellular layers, is the outermost part of the eyeball and frequently injured by external physical, chemical, and microbial insults. The epithelial-to-mesenchymal transition (EMT) plays a crucial role in the repair of corneal injuries. Zinc finger E-box binding homeobox 1 (ZEB1), an important transcription factor involved in EMT, is expressed in the corneal tissues. It regulates cell activities like migration, transformation, and proliferation, and thereby affects tissue inflammation, fibrosis, tumor metastasis, and necrosis by mediating various major signaling pathways, including transforming growth factor (TGF)-β. Dysfunction of ZEB1 would impair corneal tissue repair leading to epithelial healing delay, interstitial fibrosis, neovascularization, and squamous cell metaplasia. Understanding the mechanism underlying ZEB1 regulation of corneal injury repair will help us to formulate a therapeutic approach to enhance corneal injury repair.

## Background

**Fig. 1:**
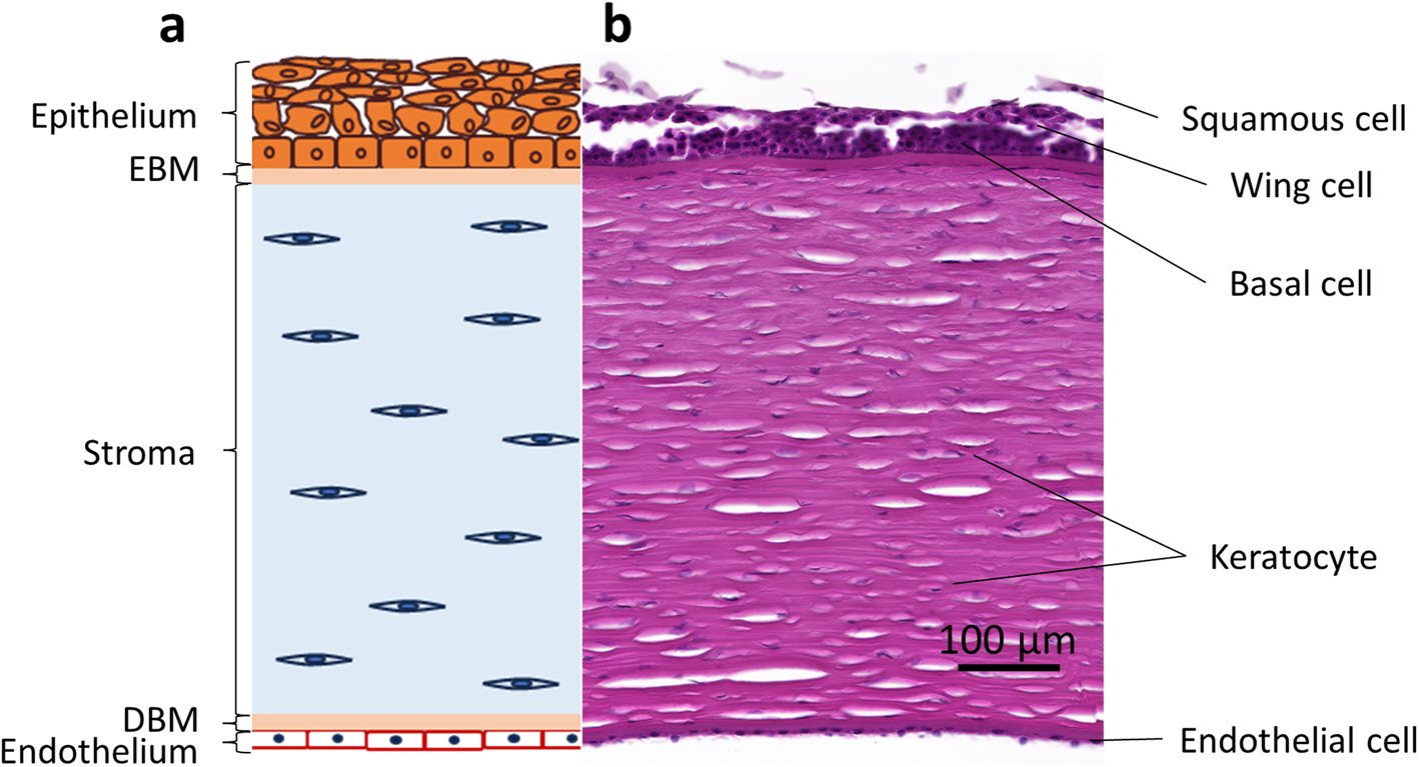
A schematic diagram of the (**a**) human cornea and (**b**) its cell types. EBM, epithelial basement membrane; DBM, Descemet’s basement membrane

The cornea is the outermost part of the eyeball and serves as a barrier against physical, chemical, and microbial insults. It is a transparent avascular tissue with biomechanical and optical properties that plays an important refractive role in the neurol imaging system [1]. The adult cornea is of an oval shape with a horizontal diameter of 11 to 12 mm and a vertical diameter of 10 to 11 mm. The central corneal thickness is about 500 μm, and the peripheral corneal thickness is about 650 μm [2]. The cornea gradually becomes flat from the center to the periphery, forming an aspherical optical system. The corneal refractive property is powerful and accounts for approximately 2/3 of the eye’s refractive power. The cornea is histologically divided into three cellular and two acellular layers. The cellular layers include the epithelium, the stroma and the endothelium containing different types of specialized cells (Fig. 1). The acellular Bowman's layer is the epithelial basement membrane (EBM) under the epithelium whereas the acellular Descemet's layer is the endothelial basement membrane (EnBM). Both membranes are composed of cell-free collagen tissue accounting for approximately 4% of the total corneal thickness [3]. The regular arrangement of collagen fibers and the uniform distribution of corneal cells and extracellular matrix (ECM) proteins are key histological features that maintain the nonlinear mechanics and transparency of the cornea [2, 4]. The majority of the corneal tissues is the stroma accounting for 90% of the thickness of the cornea and is rich in collagen. The arrangement of collagen in the anterior stroma contributes to the formation of a tighter cohesive strength in this region. Once stromal edema occurs, it affects the response of the cornea to stresses and shear forces. The tissue folds more easily in the rear when the stroma is hydrated. Therefore, the hardness of the anterior stroma is particularly important for maintaining corneal curvature [5, 6].

## Main text

### Animal models of corneal injury repair

To study corneal injury repair and its underlying mechanisms, many animal models have been created and utilized; rodents, rabbits, and pigs are the most commonly used. These model animals have a very similar anatomic eye structure compared with humans but with no Bowman’s membrane [7]. Among them, the mouse has the smallest eye and cornea whereas the pig and rabbit have the largest and median sized eye and cornea, respectively. Compared to the mouse model, rabbit and pig models are more suitable for anatomical and thereby preclinical studies though with much higher costs. The mouse is the most often used mammalian model for corneal damage repair study because of its small size, affordable cost, easy breeding, availability of ample genetic mutants and research reagents. The mouse model has been widely used for studying ocular surface diseases and various pathological mechanisms, including corneal epithelial repair [8, 9], dry eye diseases [10, 11], and targeted therapeutic treatments [12]. The widespread availability of transgenic mutants or knockout strains, particularly those conditional knockout strains where a particular gene such as Zeb1 can be deleted in a specific cell type of adult animal [13, 14], makes the mouse a very attractive model for studying corneal disease [15]. Investigations incorporating both genetic defects and environmental factors in corneal pathogenesis in mice represent a significant advancement in clarifying fundamental molecular mechanisms since many such cascades can be involved in ocular surface disorders [10, 16, 17]. The main downside of rodent models is the lesser amount of corneal tissue harvested for testing because of its small size.

### Corneal injuries and their repair mechanisms

When the corneal epithelium is damaged, it can be repaired quickly if the limbal epithelial stem cells (LESCs) are not affected [18, 19]. However, if a deeper corneal injury involves the stromal layer, it will seriously disturb the normal corneal healing process and likely result in the formation of ECM abnormalities, epithelial keratinization, stromal fibrosis, nonlinear mechanics modification, and corneal opacification [18–21]. The endothelium is the innermost layer of the cornea and plays a key role in maintaining a relatively dehydrated state of the tissue and in keeping the cornea transparent. Once the corneal endothelium is injured, its barrier may be disrupted to give rise to corneal edema and opacity [22]. Corneal opacification is the third leading cause of blindness among working-age people worldwide [23], and subsequently a corneal transplantation is required for replacement of the impaired cornea.

#### Corneal epithelial injury and repair

The tear film is the primary protective layer on the corneal surface, preventing destruction from environmental insults. It can also provide various cytokines and growth factors for the corneal epithelial homeostasis. The corneal epithelium consists of four to six layers of non-keratinized, squamous epithelial cells, including basal, pterygoid/wing, and superficial cells based on their location and shape (Fig. 1). The tight connections between the most superficial epithelial cells help maintain the barrier function of the epithelium, but only the basal cells preserve their mitotic capability [24]. Corneal epithelial cells have an average life span of four to seven days – they undergo aging (senescence), apoptosis (the programmed cell death) and shedding in an orderly manner to complete the periodic replacement of the surface cells by the basal cells. In general, the dynamic refreshing process of the corneal epithelium is completed by three following dimensions: the proliferation of the basal epithelial cells is represented as “X”, the centripetal movement of peripheral cells is represented as “Y”, and the shedding of the superficial cells from the corneal surface is represented as “Z”. By maintaining the balance of these XYZ processes, the epithelium remains stable and healthy [25].

Various physical and chemical damages on the corneal epithelium could lead to stromal swelling, inflammatory cell infiltration, fibroblast activation, and thereby loss of tissue integrity and transparency. Rapid repair of the corneal epithelium after injury is therefore crucial for maintaining corneal transparency. Corneal epithelium repair is a complex biological process that includes cell migration, proliferation, adhesion, and cell differentiation. In the first stage of the healing after a limited injury, cell migration occurs, in which intact epithelial cells adjacent to the wound do not depend on their mitosis, instead they make a single layer of cells completely recover the defect area through a cell sheet migration, while epithelial cells far away from the wound undergo mitosis, thus providing new epithelial cells to replace those migrating to the defect area [24, 26, 27]. In the second stage, cell proliferation starts, and the thickness of the epithelium returns to the normal level after the single layer of epithelial cells recover from the defect area; but the newly formed epithelium does not completely restore to a well-differentiated state. In the third stage, newly reproduced cells begin to differentiate several weeks late after the injury, the surface of the repaired area eventually becomes smooth while the defected cornea restores its well-layered structure [28]. During this entire process, multiple biological mechanisms are activated at the wound site including some growth factor/cytokine and ECM signaling pathways that mediate macromolecule interactions to restore epithelial integrity and re-establish normal corneal homeostasis [29].

In the process of corneal epithelial cell regeneration and epithelium repair, the epidermal growth factor (EGF) is the main cytokine to mediate cell migration, proliferation, and wound healing [30]. In the early stage of the epithelial wound healing, the EGF receptor tyrosine kinase EGFR1/ErbB1 activates its downstream effectors for cell signaling such as the PI3K-Akt axis, the extracellular regulated kinase, ERK, and NF-κB pathways [31, 32]. These signaling pathways play an important role in promoting corneal epithelial regeneration and wound healing. The hepatocyte growth factor (HGF) is mostly secreted by mesenchymal keratocytes, and it targets the corneal epithelial cell surface receptor c-Met in a paracrine manner. HGF is expressed in corneal epithelial, stromal, and endothelial cells, particularly in the central corneal stromal cells. HGF could regulate corneal epithelial cell proliferation, migration, and apoptosis [33]. After the corneal epithelium is injured, the expression of HGF is upregulated in both keratinocyte and epithelial cells, which may contribute to epithelial wound healing [34]. During corneal epithelial wound healing, the keratinocyte growth factor (KGF), also known as fibroblast growth factor-7 (FGF-7) secreted primarily by stromal keratocytes, activates the mitogen activated protein kinase MAPK and PI3K/p70 signaling cascades in corneal epithelial cells to promote their proliferation [35, 36].

The transforming growth factor (TGF)-β, also plays an important role in corneal epithelial repair. The isomeric TGF-β1, TGF-β2 and TGF-β3 and their receptors are all expressed in corneal epithelial cells and stromal keratocytes [37, 38]. TGF-β1 and TGF-β2 are found to inhibit proliferation of corneal epithelial cells by antagonizing EGF, HGF and KGF, but they can stimulate the proliferation of corneal stromal keratocytes [37, 38]. Studies have shown that TGF-β3 promotes wound healing of the corneal epithelium in rodents through the SMAD and PI3K-Akt signaling pathways with its target gene being PAI-1/Serpine1. At the same time, TGF-β3 can inhibit the activity of TGF-β1 and TGF-β2 to reduce scar formation in the stroma [39]. TGF-β can also stimulate corneal epithelial cell migration by regulating integrin β1 to mediate p38 MAPK activation, ECM expression, and epithelial-to-mesenchymal transition (EMT) [39].

After corneal epithelium injury, corneal inflammatory response is a necessary condition for wound healing. When the inflammation process is dysregulated by too little or too much expression of inflammatory factors, it could lead to a difficult healing process. The interleukin (IL) family plays a vital role in inflammation and in repair of corneal epithelium. Studies have shown that epithelial cells and/or immune cells are stimulated by the corneal epithelium injury to secrete IL-6, IL-10, and other cytokines to promote wound healing [40, 41]. IL-1 secreted by injured epithelial cells not only participates in the epithelial repair, but also can enter the stroma to regulate apoptosis of corneal stromal cells [29, 42]. In addition, studies have shown that IL-6 is elevated as an inflammatory cytokine in tears of patients with dry eye [40], whereas IL-10 is elevated as an anti-inflammatory cytokine in patients with rejected corneal transplant [41], suggesting that the regulation of inflammatory cytokines in corneal repair may be bidirectional. It has been identified that IL-20 plays a direct role in promoting corneal wound healing as an inflammatory factor that negatively regulates neutrophil and platelet infiltration [43]. It is known that the small molecule retinoic acid improves corneal wound healing [44] likely through stimulating NF-κB [45], a transcription factor involved in regulating pro-inflammatory cytokine gene expression of factors such as tumor necrosis factor (TNF)-α and IL-1β.

In addition to cell proliferation, adhesion of epithelial cells to the ECM also plays a significant role in repair of corneal epithelium injury [28, 46]. The basal ECM of the corneal epithelium is a specialized EBM [29]. Type I collagen is the main component of the ECM, constituting an extracellular space to provide basic support for epithelial cells. Other components such as Type IV collagen, laminin, and fibronectin, are involved in regulating biological functions of corneal cells, including induction of corneal epithelial cell differentiation [47, 48]. The adhesion of corneal epithelial cells to the ECM is mediated by integrins on the cell surface membrane. Integrins are composed of heterodimers of α and β subunits, each of which contains an extracellular domain responsible for ligand binding, a single transmembrane domain, and a cytoplasmic domain. There are at least 24 different heterodimers with different binding specificity to ECM components, and multiple integrin heterodimers are expressed in the corneal epithelium [49]. In human and mouse central corneas, integrins α2β1, α3β1, αvβ5, and α6β4 all show polarized localization in the epithelium, they are highly expressed in basal cells but not in the apical squamous epithelium [49, 50]. Integrins expressed on the EBM can mediate adhesion of epithelial cells to ECM components like collagen, fibronectin, laminin, and etc. Epithelial cell migration on the EBM depends on the cycle of adhesion and deadhesion between the cells and ECM substrates. If the adhesion to ECM proteins is too strong, the epithelial cells may not be able to move. When the expression of integrin decreases, the epithelial cells reduce their proliferative ability and decrease their adhesive ability to the EBM [28]. The bidirectional communication between epithelial cells and the ECM is called "dynamic reciprocity" and mediated by biophysical signals and mechanical transduction pathways. Integrins can transmit biomechanical signals from the cytoplasmic domain to the cytoskeleton and then to the nucleus to regulate gene expression, they play an essential role in maintaining tissue integrity [51].

The EBM of 0.05 mm thick is a highly specialized ECM and mainly composed of laminins, collagens, heparin sulfate proteoglycans, and nidogens secreted by the epithelial basal cells [52]. If the level of damaged fibronectin increases, the healing process may take up to six weeks. During this time however, the adhesion of epithelial cells to the newly repaired EBM is unstable [5]. The EBM has been examined in detail under a transmission electron microscope, and it is often identified as two adjacent layers, called lamina lucida and lamina densa [53]. Although the stratification may be caused by an artifact of electron microscopy, the existence of two layers is considered to be the in situ of the normal EBM [53]. The regeneration of the EBM after injury is a self-assembly process mediated by surface adhesion, inter-component binding, and polymerization. Laminin is an essential promoter for EBM formation because of its ability to bind to other laminins, EBM components, and cell surface molecules [54]. When an epithelial repair is initiated, laminin scaffolders can recruit and assemble remaining EBM components, including nidogen-1, nidogen-2, perlecan and type IV collagen to achieve structural and functional maturation of the regenerated EBM [55]. The structure and function of the EBM are important in regulating the bidirectional transmission of cytokines. The EBM can regulate not only the entry of EGFs and cytokines like TGF-β1 and platelet-derived growth factor (PDGF) to the stroma, but also the effect of related cytokines and growth factors such as KGF on epithelial cells [56]. In summary, the integrity of the EBM has a profound impact on the corneal injury repair process, which is not only reflected in the healing of the corneal epithelium, but also involved in the regulation of corneal stroma repair.

#### Damage and regeneration of limbal epithelial stem cells (LESCs)

The corneal epithelium is constantly worn off and regenerated due to the homeostasis of the tissue and damage repair by a unique group of unipotent stem cells at the limbus, the border between the cornea and the sclera, known as LESCs in the basal layer of the epithelium at the margin of Vogt [57]. The limbal location of LESCs and LESC migration to the central cornea are confirmed by single-cell tracking technology which is in tandem with LESC hypotheses [58, 59]. LESCs have a long-lasting self-renewal ability throughout their lives and maintain a constant number while producing rapidly dividing progenitor cells known as transit amplifying cells (TACs) [60]. These TACs, which make up most of the basal epithelium in the limbus and in the peripheral cornea, can migrate to the central cornea, rapidly proliferate, and differentiate into the central corneal epithelium. This epithelial regeneration occurs during corneal homeostasis and injury repair [60, 61]. The regeneration of every structural compartment in a wounded cornea seems to involve different mechanisms and cell types. With the latest single-cell technology, the quantitative genealogy of LESCs in the limbal epithelium of mice has been tracked; there are two populations of LESCs located in different regions. LESCs located outside the limbus are static and regulated by T cells, and mainly involved in wound healing and boundary formation whereas those LESCs located inside the limbus are dynamic in maintaining corneal epithelial homeostasis [62]. Inherited or acquired deficiency of LESCs, clinically known as limbal stem cell deficiency (LSCD), may lead to serious corneal disorders like delayed corneal wound healing, interstitial neovascularization (NV), and conjunctival cell ingrowth, ultimately resulting in corneal opacity and visual loss if not treated properly [63]. Thus, cytokines or growth factors that activate LESCs would benefit corneal epithelial wound healing. For example, the cytokine KGF that is required for activating LESCs, was reported to play an important role in corneal epithelial wound healing [64]. In addition, the exogenous neuroprotective factor—ciliary neurotrophic factor (CNTF) promotes corneal epithelial wound healing also by activating LESCs as its neutral antibodies can delay wound healing [65]. Although studies have shown that some growth factors/cytokines and chemotactic molecules are involved in the corneal epithelial wound healing, the initiation signaling mechanism of the LESC/TAC activation, proliferation and migration is still not fully understood. Interestingly, Notch1 has been identified to be essential for corneal epithelial repair after injury [66], and its related signaling is required for corneal epithelial differentiation [67].

Corneal sensory innervation plays a critical role in the corneal epithelial homeostasis and wound healing, its impairment due to corneal injury would reduce both protective reflexes and trophic neuromodulators, thereby negatively affect wound healing of ocular surface tissue and may lead to neurotrophic keratitis [68]. The cornea is innervated by sensory nerves, the axons in the basal epithelial layer of the limbus run adjacent to the LESCs, and their free nerve endings contact epithelial cells [69]. Accumulating evidence support a critical role of corneal nerves in regulating epithelial renewal by exciting the activity of LESCs [69]. It is of note that the corneal axons interact with Schwann cells (SCs), and SCs in the nerve terminals facilitate tissue regeneration through secreting the neurotrophic nerve growth factor (NGF) [70]. NGF acts synergistically with other trophic factors such as CNTF, PDGF-α, and TGF-β, to regulate the activity of LESCs [69]. Although there has been some progress in fundamental understanding, the regulatory mechanisms of LESC proliferation and differentiation remains mostly unknown.

#### Corneal stromal damage and repair

The corneal stroma occupies approximately 90% of the cornea. Its well-organized collagenous structure with the precise arrangement of stromal fibers makes its ECM extremely transparent. The collagen fibers, also known as fibrils, are packed in parallel as well-arranged lamellae. The stroma of the human cornea contains 200 to 250 distinct lamellae [71]. The packing density is higher in the anterior stroma than in the posterior stroma [72]. The anterior and mid-stromal lamellae are highly interwoven, while the posterior lamellae are less interwoven and more hydrated [73, 74]. Thus, the posterior stroma can swell easily whereas the more interwoven anterior cannot [75]. As a main load-bearing component of the lamellae, collagen fibrils should not only resist the tension generated by the intraocular pressure and protect the intraocular tissues from external trauma, but also ensure the regular arrangement of the fibers to reduce light scattering and interference and to maintain the transparency of the tissue.

Corneal stromal wounds may be a result of traumas and refractive surgeries to correct ametropia. It should be noted that the wound healing occurs upon stromal damage. Fibrotic scar formation because of wound healing could reduce corneal transparency and thereby compromise vision. The stromal scars would disappear upon proper healing as the stroma can undergo precise remodeling with its orderly arrangement of collagen lamellae to re-ensure corneal transparency without NV. This process is a result of the interaction of autocrine and paracrine cytokines, growth factors, chemokines secreted by epithelial cells, stromal cells, bone marrow-derived cells and nerve cells, and their receptors on the stromal cells for reconstruction of normal corneal structure and for regaining normal tissue function [76]. The initial appearance of keratocyte apoptosis in the anterior region is an indication of a start of corneal stromal wound healing. Release of inflammatory cytokines by epithelial cells and/or tear fluid, i.e., IL-1 (α and β), through the Fas–Fas ligand system leads to a rapid apoptosis [77, 78]. After the initial wave of keratocyte death, some parts of the corneal stroma may undergo necrosis, resulting in more serious tissue inflammation and destruction [79]. In general, 12 to 24 h after a corneal epithelial injury, the activated keratocytes in the surrounding and posterior stroma begin to proliferate and migrate [80]. At this time, keratocytes in the apoptotic and necrotic areas were reported to be Ki67^+^ (a marker of cell proliferation) [80]. Keratocytes can transdifferentiate into fibroblasts and myofibroblasts, proliferate, and migrate to the injured site [81, 82]. Meanwhile, bone marrow-derived cells from blood vessels in the limbus migrate to the stroma. Studies have shown that up to 70% of myofibroblasts in the corneal stroma are developed from bone marrow-derived precursor cells [83]. In addition, through the process of EMT or endothelial to mesenchymal transition (EnMT), keratocytes may also be the origin of myofibroblasts in some cases [84].

Activated myofibroblasts contain intracellular α-SMA^+^ filaments with high mobility and strong contractility in the corneal stroma, which can promote corneal wound healing and restoration of corneal integrity [85]. They deposit and crosslink excessive ECM proteins, including collagen and fibronectin, causing destruction of normal tissues [86]. Corneal myofibroblasts also express fibronectin, α5β1, and αvβ3 integrin receptors, which connect the actin cytoskeleton to the ECM, enabling them to contract and remodel the wound [87]. Crystallin is a water-soluble protein found in the lens and in the cornea, accounting for the transparency of the structures. Fibroblasts downregulate the expression of corneal crystallin, resulting in persistent stromal haze, and upregulate expression of matrix metalloproteinases (MMPs) to remodel the wound ECM for repair [88]. As mentioned above, once the cornea is injured, corneal cells can express many different growth factors and cytokines such as FGF, KGF, PDGF, EGF, TGF-β, Insulin-like growth factor (IGF), HGF, TNF, and IL family members. The transdifferentiation of keratocytes into fibroblasts/myofibroblasts is mediated by some growth factors like FGF2, PDGF-AB, and TGF-β. Other growth factors like IL-1 and IGF1 only stimulate the mitotic activity of target cells [89, 90]. TGF-β has been found to play a key role in generation of highly metabolically active myofibroblasts in the cornea that in turn produce more TGF-β [8]. Thus, once myofibroblasts are produced, they can maintain their own vitality through autocrine mechanisms [91]. Once wound healing is complete, overexpressed IL-1 by stromal cells triggers the apoptosis of myofibroblasts, resulting in a decrease of TGF-β [91], followed by keratocytes reoccupying the prostroma and absorbing abnormal ECM proteins to restore corneal integrity and clarity [92].

In the normal uninjured cornea, epithelial and endothelial TGF-β and PDGF production and/or activation is relatively low. These growth factors are prevented from entering the corneal stroma by the EBM and endothelial basement membrane (EnBM). When a damage occurs in the epithelium-EBM and/or the endothelium-EnBM, TGF-β, PDGF, and possibly other growth factors and cytokines enter the corneal stroma at higher levels, triggering surviving keratocytes to transform into corneal fibroblasts, and these fibroblasts then begin to develop into mature myofibroblasts [42]. However, any persistence of TGF-β maintains the activity of myofibroblasts, which will continue to secrete and deposit abnormal ECM, resulting in corneal cloudiness that persists long after myofibroblasts have disappeared from the injury site [85, 88].

#### Corneal endothelium injury and repair

The corneal endothelium is the posterior layer of the cornea with a thickness of about 4 μm in adults. It is composed of a monolayer of hexagonal corneal endothelial cells (CEnCs) derived from the neural crest [93]. As a barrier between the corneal stroma and the anterior aqueous humor chamber, the corneal endothelium has extensive tight junctions, consisting of adhesion proteins like zonula occludens (ZO-1) and connexin-43 and adhesion junction complex like cadherin isomers. The lateral membranes of CEnCs contain a high-density Na^+^ /K^+^ -ATPase pump, which can control the influx of water, ions, and metabolites. This water pump can promote the flow of fluid from the corneal stroma of high osmosis to the aqueous humor of low osmosis in the anterior chamber of the eye. Therefore, CEnCs play an essential role in maintaining the relative dehydrated state of the corneal stroma (78% water content) and the correct arrangement of stromal collagen [94–97]. The basal surface of the corneal endothelium contains many hemidesmosomes that promote the adhesion of CEnCs to the basal membrane EnBM [5].

CEnCs are usually arrested in the G1 phase of the cell cycle and cannot be regenerated by themselves due to their highly differentiated status. Once damaged, the endothelium conducts its repair through cell migration and an increased diffusion; CEnCs thereby exhibit high polymorphism [98]. The cell density of human CEnC at birth is approximately 3500 cells/mm^2^, and this density in healthy individuals gradually decrease over time, with an average loss of about 0.6% per year [99]. When it is below 500 cells/mm^2^, the endothelial water pump function may fail and there is a risk of corneal edema [100]. In this case, the most effective treatment is the corneal endothelial transplantation, which replaces the damaged endothelium with a functional one.

The injury to the corneal endothelium can be divided into two categories: direct injury such as alkali burn, corneal transplantation, and cataract surgery, and indirect injury that is caused by the apoptosis and regeneration of epithelial cells, stromal cells, and endothelial cells and affected by corneal wound healing [101]. The migration of CEnCs depends on the formation of filopodia and lamellipodia and is related to the dynamic change of their cytoskeletal actin filaments [102]. During corneal endothelial wound healing, ECM components such as fibronectin and laminin affect CEnC migration by regulating the reorganization of the cytoskeleton and the migration direction of the cells along the EnBM [103]. Healthy CEnCs undergo cytoskeletal changes during wound healing, including actin recombination and cell enlargement, forming a polygonal cell shape to recover the damaged area and thereby restore the barrier function of the endothelium. These cell phenotypic changes are also known as EnMT [104]. The hallmark of EnMT is the downregulation of the E-cadherin adhesion protein and upregulation of cytoskeletal proteins such as fibronectin and vimentin, which is accompanied by an increased gene expression of type I collagen [105]. Once EnMT occurs, CEnCs will lose their normal shape and function due to the loss of their tight junction protein ZO-1, destruction of the cell monolayer, and transformation into an α-SMA^+^ myofibroblast-like cell phenotype. These transformed cells escape from neighboring cells and migrate to the defect area along the EnBM to facilitate rapid wound closure [106–108]. EnMT and fibrosis of CEnCs during the wound healing are either directly or indirectly regulated by a variety of cytokines, including FGF-2, PDGF-BB, IL-1β and TGF-β. Unfortunately, the mechanism of corneal endothelial cell regeneration is not as clear and requires further investigation [98].

### ZEB1 regulation of corneal injury repair

#### General introduction of ZEB1

The Zinc finger E-box binding homeobox (ZEB) transcription factor family consists of two members, ZEB1 and ZEB2 [109]. The cDNA sequence of human ZEB1 gene consists of 217,642 bp that can be transcribed into 32 different mRNA variants. The ZEB1 protein contains 1117 amino acids with both zinc finger clusters at the N- and C-terminals that can bind to the specific DNA consensus sequence CANNTG, also known as E-box, of target genes to regulate cell differentiation and tissue-specific functions [110, 111]. ZEB1 primarily serves as a transcription suppressor by interacting with SMAD proteins and C-terminal binding protein (CtBP) to form histone deacetylase complexes on gene promoters/enhancers. The formation of the ZEB1-CtBP complex is essential for the downregulation of genes like E-cadherin (*CDH1*), mucin 2, and platelet proteins [112]. ZEB1 is also involved in cell senescence and apoptosis [113]. By reducing the expression of CDH1, ZEB1 stabilizes the distribution of β-catenin in the nucleus, strengthens the Wnt signaling, leading to cell cycle arrest at the G1 phase [114]. ZEB1 is a major regulator of EMT that promotes cell proliferation, differentiation, and migration, and plays important roles in development, cancer progression, and tissue fibrosis [115]. CDH1, a cell adhesion molecule with two independent C2H2-type zinc finger domain arrays and a central homologous domain, exists in the plasma membrane of normal epithelial cells. Downregulation of CDH1 is a hallmark of EMT [116]. EMT plays an essential role in normal tissue development such as the formation of the gastric neural crest, cardiac, musculoskeletal, and craniofacial structures [117]. Some studies have shown that ZEB1 can act as an activator. For example, ZEB1 binds to two sites in the vitamin D promoter in vitro to directly activate the transcription of this receptor [116]. The cellular environment also plays a crucial role in determining whether ZEB1 functions as a transcriptional suppressor or an activator. For example, in MDCK cells, ZEB1 activates the transcription of the ATPase 1 promoter, while in rat fibroblasts, ZEB1 displays an inhibitory role [118].

ZEB1 is expressed in the corneal epithelium, stroma, and endothelium [119]. In recent years, numerous groups have proposed that ZEB1 dysfunction may disrupt the homeostasis of corneal epithelial stem cells, leading to corneal cell apoptosis, stromal fibrosis, angiogenesis, and squamous metaplasia [120]. Interestingly, the intrinsic factor lumican, a small leucine-rich proteoglycan (SLRP) in the cornea, has shown anti-cancer activities by suppressing ZEB1 [121]. As an upstream factor of ZEB1 and a MET marker [122], lumican is therefore critical for corneal epithelial wound healing [123]. Understanding how ZEB1 regulates the development and progression of these diseases will help us to identify potential targets for diseases associated with ZEB1 overexpression.

#### ZEB1 regulation of corneal inflammation

Studies have shown that Zeb1 is widely expressed in immune cells in the bone marrow and plays a crucial role in T cell differentiation [124]. In mice, Zeb1 may regulate T cell development by repressing genes containing the E-box like *Il-2, Cd4, Gata3*, and *Itga4*. For example, Zeb1-deficient neoplastic dendritic cells stimulated by the toll-like receptor 9 (TLR9) agonist CpG may cause T cells to differentiate into Th2 instead of Th1 [125]. In Zeb1-deficient mice, the size of the thymus and the number of mature CD4^+^ T cells were significantly reduced [126]. The C-terminal zinc finger of ZEB1 inhibits *Il-2* gene expression in Th2 cells by binding to the negative regulatory element NRE-A in the *Il-2* promoter [127]. Naive CD8^+^ T cells express high levels of ZEB1, which decreases after T cell activation and then becomes upregulated again in memory CD8^+^ T cells [128]. Zeb1-deficient mice exhibited impaired expansion of CD8^+^ T cells, reduced expression of cytotoxic granzyme B, and decreased control of bacterial load [128]. These findings suggest that ZEB1 plays a role in the establishment, maintenance, and execution of specific functions of memory CD8^+^ T cells and the maintenance of normal protective immunity [129].

**Fig. 2:**
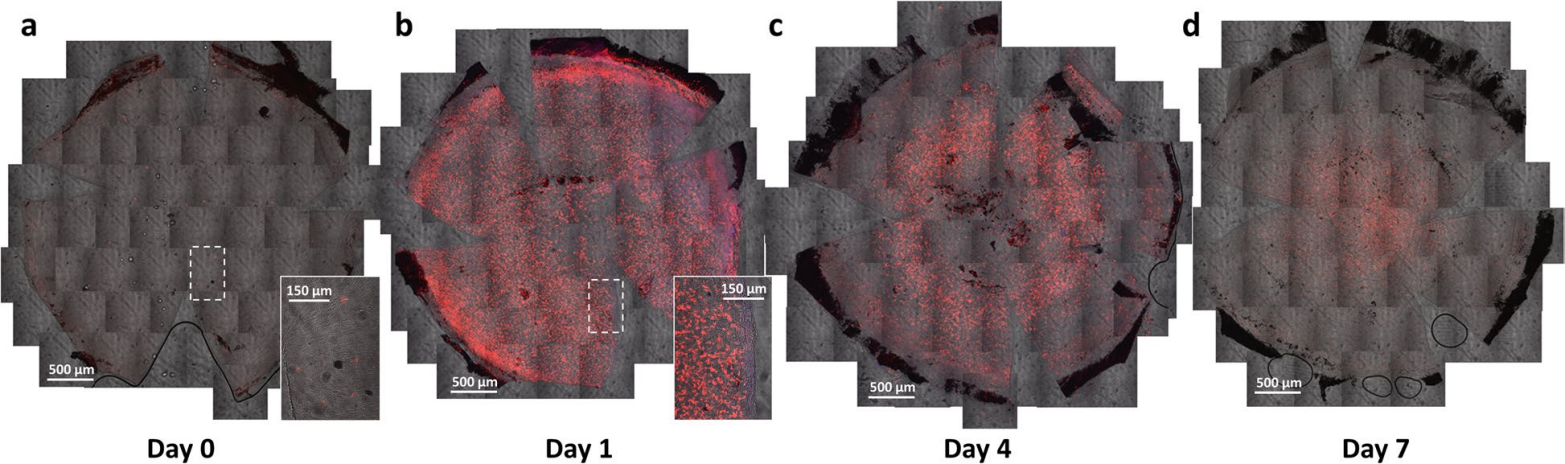
Influx of Lyz2^+^ immune cells to the central cornea following alkali burn. a Very few Lyz2^+^ cells are scattered over the entire cornea before an alkali burn. **b** An immediate accumulation of Lyz2^+^ cells in the cornea at 1 day after the alkali burn. **c** On Day 4 after the alkali bure, more Lyz2^+^ cells remain in the precentral area around the wound site. **d** On Day 7 after the alkali burn, most accumulated Lyz2^+^ cells have disappeared except for those in the central area

To demonstrate the dynamic change in the immune cell infiltration into the cornea after an alkali burn, we bred transgenic CAG-STOP^f/f^-tdTomato mice [130] with Lyz2-Cre mice [131, 132] to create a CAG-STOP^f/f^-tdTomato; Lyz2-Cre^+^ mouse corneal inflammation model where Lyz2^+^ myeloid cells is specifically tagged by the red fluorescent tdTomato. Before alkali exposure, very few Lyz2^+^ cells were scattered over the avascular cornea although more Lyz2^+^ cells were found in the limbal area where the vasculature exists, suggesting that tdTomato^+^ (i.e., Lyz2^+^) cells are resident myeloid cells, likely local macrophages as reported (Fig. 2a) [133]. After alkali burn, there was an immediate accumulation of more Lyz2^+^ cells in the damaged cornea where four distinct zones exists: (1) the limbal area, (2) the perilimbal area, (3) the pericentral area and (4) the central area (Fig. 2b). It seems like most circulating myeloid cells begin to migrate from the limbus, through the perilimbal zone, with more staying in the pericentral zone and even fewer moving to the central area on the first day following alkali burn (Fig. 2b). In four days, more existing Lyz2^+^ cells died compared with the inflowing cells (Fig. 2c). In seven days, most Lyz2^+^ cells fainted away except for those in the central cornea (Fig. 2d), indicating the location where the immune cells accumulated is consistent with the location where the epithelial was recovering. It appeared that the alkali burn induced an accumulation of Lyz2^+^ cells around the corneal wound site which are required for corneal epithelial wound healing.

It is known that the basal membrane EBM of the corneal epithelium stores significant amounts of inflammatory cytokines, which can be rapidly released at the injured epithelium site; their release initiates an inflammatory signal in the surrounding stroma and attracts immune cells to the site [42]. In an alkali-induced mouse corneal NV model, a critical role of Zeb1 in cell proliferation has been demonstrated [134]. It was observed that Zeb1 is expressed at high levels in corneal epithelial basal cells, vascular endothelial cells, and infiltrated immune cells [134]. ZEB1 is known to bind to and transactivate a group of inflammatory cytokine genes, including *Il-1b*, *Il-6*, *Il-8*, *Tnfa*, and *Csf2* [135]. Another study discovered that ZEB1, and CREB bind to the promoter elements of pro-inflammatory cytokines *Il-1b* and *Ifn-γ*, and could upregulate their expression in corneal epithelial cells through the p38 MAPK-mediated signaling pathway [136]. This study identified ZEB1 and CREB as the key intracellular signaling intermediates in regulating the immune-mediated process of ocular surface squamous epithelial metaplasia [136]. To examine the potential impact of inflammatory cytokines on Zeb1 and vice versa in the cornea, we utilized alkali burned corneas to induce both corneal inflammation and NV [134, 135]. We provided evidence that Zeb1 was indeed involved in regulating inflammation and NV in the cornea [135].

#### ZEB1 regulation of corneal epithelial wound healing

ZEB1 promotes EMT, and upregulation and activation of ZEB1 are positively correlated with cell proliferation and migration because it inhibits expression of the epithelial gene *CDH1* [137]. As previously mentioned, corneal epithelial basal cells, a mitotic population, are regenerated by LESCs and therefore express the cell proliferation marker Ki67 [138]. In normal mouse corneas, nuclear expression level of Zeb1 in epithelial cells is low, even though LESC-derived basal cells continue to divide [5, 139]. Our subsequent studies on the mechanically debrided corneal epithelium in mice found that monoallelic knockout of Zeb1 (Zeb1^+/−^) resulted in the partial loss of Zeb1 expression in the corneal epithelium and reduction of the vitality and mobility of corneal epithelial cells, and thereby delayed the recovery of the debrided epithelium [138]. In the first three days after debridement, there was no significant difference in the number of Ki67^+^ cells between Zeb1^+/+^ and Zeb1^+/−^ corneas. However, the proliferation rate of Zeb1^+/+^ corneal epithelial cells increased significantly on day seven and beyond, while no such increase was found in the Zeb1^+/−^ cornea [138]. This suggests that the monoallelic Zeb1-knockout only reduced corneal epithelial cell proliferation in the later stages of the wound healing. Upon further study, we discovered that Cdh1 was highly expressed in basal cells along the corneal epithelium before the debridement, while no Vim was detected. After six hours, corneal epithelial cells expressed Vim before the wound, while these cells expressed Cdh1 after the wound, suggesting that EMT occurred only at the leading edge of the epithelial wound. However, this EMT was short-lived and immediately reversed when these mobile epithelial basal cells returned to the denuded area within 1 day after the debridement, indicating that a distinct switch in EMT in corneal epithelial cells is under the control of Zeb1. To facilitate cell movement, corneal epithelial cells require not only EMT but also additional ECM-degrading enzymes such as MMPs and urokinase PLAU to release epithelial-matrix binding, more ECM proteins such as fibronectin (FN)1 to replace the degraded ECM, and increased cell matrix anchors such as integrins such as integrin α5 to rebuild their binding to the ECM and to promote corneal epithelial wound healing [138]. In summary, ZEB1 may regulate the movement of corneal epithelial cells by inducing transient EMT, degrading cell–matrix connections, renewing the ECM, and re-establishing new cell-ECM connections.

#### ZEB1 regulation of corneal neovascularization (NV)

ZEB1 is an EMT factor essential for embryonic development [140]. Studies have found that murine embryos with homozygous Zeb1 knockout (Zeb1^−/−^) die before birth due to respiratory failure in the perinatal period and exhibit a variety of bone defects, including craniofacial deformities, limb and sternal defects, rib deformities, and severe T-cell deficiency in the thymus [126, 141]. Zeb1^−/−^ embryos first exhibit small bleeding or vascular malformations in the head at embryonic day (E)11.5 [140]. The defects became more pronounced at E15.5, manifesting developmental retardation, edema, multiple bleeding, and curled legs and tails [140]. The cornea is an avascular tissue, a lasting inflammation in the cornea depends on new blood vessel formation known as NV, a serious medical condition that may lead to reduced vision and even blindness. In the normal healthy cornea, many cytokines inhibit NV that often requires a hypoxic condition. The cornea is however not exposed to a hypoxic environment, and all nutrients and oxygen that corneal cells rely on are from infiltration of both tear fluid outside the epithelium and liquid in the anterior chamber behind the endothelium. Therefore, NV after corneal injury is not caused by hypoxia; but instead, promoted by the cytokines secreted by corneal cells [77]. After injury, the injured corneal epithelium, basal membrane EBM and corneal stromal cells start releasing large amounts of inflammatory cytokines and matrix metalloproteins [77], leading to the apoptosis of epithelial and stromal cells and promoting an infiltration of neutrophils and monocytes/macrophages into the corneal stroma [142]. These immune cells in turn also secrete large amounts of cytokines and cell growth factors including vascular endothelial growth factor (VEGF), EGF and FGF which further promotes corneal NV [77, 142]. VEGF is the most potent cytokine that fosters tissue angiogenesis. In normal healthy eyes, corneal cells can neutralize VEGF to prevent corneal NV by expressing the soluble VEGF receptor, VEGFR-1 [143].

The development of new vasculature depends on the proliferation of vascular endothelial cells. Zeb1 is one of the important factors that can directly participate in the regulation of cell cycle activities by inhibiting cyclin-dependent kinases (Cdk) inhibiting proteins (such as p15 and p21) [113], so it might be required for regulating the proliferation of vascular endothelial cells. Indeed, Zeb1-deficient mice mainly manifest reduced immune cells [126, 141], slow growth of organ tissues [140], abnormal capillary development [134], and accelerated cell senescence [113]. ZEB1 was also found to promote in vivo tumorigenesis and angiogenesis in breast cancer by promoting expression of VEGF [144], while Fu et al. identified that endothelial ZEB1 promoted angiogenesis-dependent bone formation through the upregulation of VEGF [145]. It appears that corneal NV can be stimulated by ZEB1-upregulation of VEGF although Jin et al. did not confirm this positive relationship between Zeb1 and VEGF-A in the mouse model of alkali burn-induced corneal NV [134]. In addition, VEGF antibodies have shown limited efficacy in treating tumors and corneal NV [146, 147], suggesting that angiogenesis is a complex process where VEGF is one of the many factors involved. It has been demonstrated that ZEB1 can indirectly promote the expression of VEGF by inhibiting miR-200b [148, 149], and then promote the generation of tumor NV [144, 150]. We recently found that the monoallelic knockout of Zeb1 significantly reduced corneal NV [134], so it is of great significance to elucidate the molecular mechanism by which ZEB1 regulates tissue NV.

ZEB1 can inhibit the expression of miR-200 family members, and miR-200 family members can also inhibit expression of ZEB1, suggesting that there is a feedback balance between these two [149, 151]. We hypothesize that the negative feedback loop between ZEB1 and miRNA upregulates the expression of pro-angiogenic factors, and thus promotes proliferation of vascular endothelial cells. In parallel, we also hypothesize that these pro-angiogenic factors promote NV by increasing ZEB1 expression and thereby promote vascular endothelial cell division. Thirdly, ZEB1 may be a completely new pro-angiogenic factor unrelated to the above known pro-angiogenic factors and has its own unique molecular regulatory mechanism. In general, ZEB1 binds to related genes such as *CDH1* by interacting with the histone deacetylase (HDAC) and CtBP to inhibit binding of RNA polymerase to double-stranded DNA which repress the expression of target genes [152]. If CtBP is separated from ZEB1, the histone methyltransferase (HMT) has the chance to interact with ZEB1 to form a new complex that makes it easier for the RNA polymerase sitting on double-stranded DNA to synthesize target genes and increase their expression. Certain cofactors are required or removed to maintain the integrity of the ZEB1-CtBP complex. For example, the small molecule nicotinamide adenine dinucleotide (NADH) helps CtBP bind to ZEB1; however, 4-methylthio-2-oxobutanoic acid sodium salt (MTOB) exhibits substrate inhibition and can interfere with CtBP activity [153]. Interestingly, NSC95397 was recently identified as a ZEB1-CtBP inhibitor [134]. Both MTOB and NSC95397 were explored for inhibition of ZEB1 activities; but only NSC95397 could repress Zeb1 expression and diminish its physiological roles [134]. Therefore, we conclude that the regulation of corneal NV by Zeb1 is independent of VEGF and the ZEB1-CtBP inhibitor, NCS95397, may have the therapeutic potential for ocular NV and possible cancers.

#### ZEB1 regulation of corneal scarring

Corneal trauma and infection that lead to inflammation and NV, followed by irreversible tissue fibrosis, are the main causes for visual damage, so to find a target protein for the treatment of fibrosis is an urgent clinical need for ophthalmologists. ZEB1 is considered an oncoprotein because it is highly expressed in many malignant tumors derived from epithelial tissues [154–156]. EMT can be activated in tumor cells, leading to abnormal cell movement, thereby triggering metastasis, and imparting mesenchymal characteristics to tumor cells [157]. It is well known that the transcription factors SNAI, TWIST, and ZEB families are the primary activators of EMT [158]. Among them, ZEB1 can induce epithelial cells to transition to a more migratory and invasive mesenchymal phenotype during development, primarily by repressing the epithelial cell marker E-cadherin (CDH1) to reduce intercellular adhesion. Concurrently, the mesenchymal cell marker N-cadherin (CDH2) is upregulated to facilitate cell migration and invasion to promote tumor progression and metastasis [113, 159]. In the case of fibrotic diseases, EMT is involved not only in the transformation of epithelial cells into mesenchymal-like cells, but also the acquisition of characteristics to enhance crosstalk between epithelial and mesenchymal cells [160]. ZEB1 can also activate various signaling pathways like Wnt, Hippo, and TGF-β signaling pathways to regulate tissue transformation, fibrosis, and necrosis [109]. The inflammatory cytokine TGF-β stimulates ZEB1 to initiate the activation of the Wnt/β-catenin signaling pathway that induces hepatic stellate cells to upregulate the expression levels of α-SMA and type I collagen. This process promotes the secretion of IL-6 and TNF-α, leading to liver fibrosis and liver cancer development [161].

The fibrosis in different organs and tissues shares common pathological events, including fibroblast activation, EMT, endothelial cell dysfunction, and immune cell polarization. These are induced through paracrine or autocrine cytokine regulatory signaling pathways [162]. In a pulmonary fibrosis disease model, Zeb1 was found to regulate parathyroid hormone signaling between pulmonary epithelial cells and fibroblasts, and to control epithelial-mesenchymal crosstalk by regulating the expression of tissue plasminogen activator (tPA) which contributes to the development of pulmonary fibrosis [163]. Multiple signaling pathways are activated following a corneal stromal injury, including the TGF-β, bFGF, and PDGF-BB pathways. Among these, the TGF-β signaling pathway plays a key role in promoting activation and proliferation of corneal fibroblasts [135]. Despite a prolonged exposure to the EMT stimulator TGF-β, ZEB1-deficient tumor cells maintained an epithelial phenotype [164], suggesting that ZEB1 may be required for the progress of EMT-related fibrosis. ZEB1 has been shown to upregulate TGF-β expression, leading to the formation of a positive regulatory loop between TGF-β signaling and ZEB1 activation [150]. Liang et al. found that Zeb1 can form a positive regulatory loop with TGF-β [135]. Normally, corneal fibroblasts are in a resting state; but once the cornea is injured, the TGF-β signaling pathway is activated [135], leading to transformation of neighboring fibroblasts into myofibroblasts expressing α-SMA. These myofibroblasts secrete abnormal ECM components such as collagen type 1 (Col1), collagen type 3 (Col3), hyaluronan, and extra domain A of fibronectin (EDA-FN), which contribute to fibrosis of the corneal stroma [165]. Another characteristic of corneal fibrosis is the ability of epithelial cells to transdifferentiate into fibroblasts through type 2 EMT during tissue repair [88, 166].

**Fig. 3:**
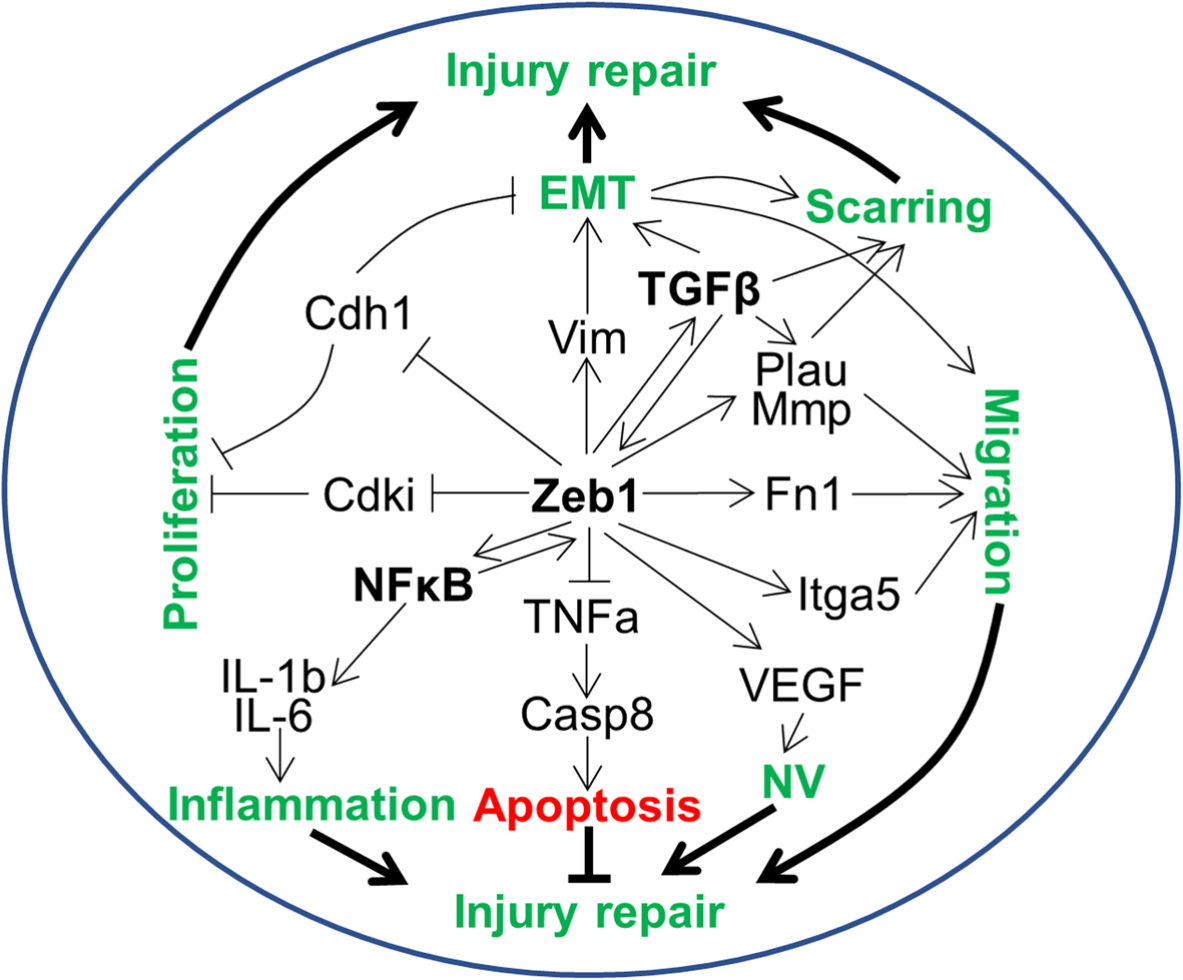
Schematic diagram to demonstrate ZEB1 regulation of many genes involved in corneal injury repair. ZEB1 is mainly regulated by TGF-β to promote EMT and likely tissue scarring. ZEB1 also upregulates the inflammation master factor NFκB and the NV master factor VEGF, and downregulates the apoptotic factor TNF-α to promote corneal inflammation and to affect corneal injury repair. Casp8, caspase 8; Cdh1, E-cadherin or cadherin 1; Cdki, cyclin-dependent kinase inhibitor; EMT, epithelial-to-mesenchymal transition; Fn1, fibronectin 1; IL, interleukin; Itga5, integrin subunit alpha 5; Mmp, matrix metalloproteinase; NFκB, nuclear factor kappa B; NV, neovascularization; Plau, urokinase-type plasminogen activator; TGF-β, transforming growth factor beta; TNF-α, tumor necrosis factor alpha; VEGF, vascular endothelial growth factor; Vim, vimentin; Zeb1, Zinc finger E-box binding homeobox 1

Multiple studies have shown that ZEB1 can also bind to microRNAs (miR-200c and miR-205) to mediate multiple signaling pathways, it is a molecular motor that influences cell plasticity through a feedback pathway involving members of the miR-200 family [117, 167, 168]. By binding to specific proteins such as SMAD, BRG1, YAP, and CtBP, ZEB1 mediates various signaling pathways [109]. Early corneal inflammation is regulated by maintaining the proliferation and migration of immune cells, while late wound healing is regulated by activating NFκB and TGF-β-related Stat3 signaling pathways (Fig. 3) [135]. For corneal injury repair, we hypothesize that the cornea may activate corneal myofibroblasts through the TGF-β pathway when serious corneal damage occurs under the stimulation of inflammatory factors. This activation may lead to secretion of abnormal ECM, and thereby changes in the structure and hardness of the corneal ECM. Corneal cells may then sense the mechanical stress of the matrix and initiate signal transduction that contributes to the corneal fibrosis process.

## Conclusions

ZEB1 is an essential factor in embryonic development and expressed in most developing tissues; complete knockout of Zeb1 in murine embryos leads to death before birth. In adults, ZEB1 is mostly not expressed in normal tissues. Damaged and diseased tissues, however, may express high ZEB1, particularly in the dividing cells of tumor and fibrotic tissues. Mounting evidence has shown that ZEB1 plays an important role in tumorigenesis and metastasis, tissue fibrosis, and wound healing, mostly through regulation of EMT of related cells. We have provided some evidence that Zeb1 affects corneal injury repair by regulating corneal cell migration, apoptosis, and proliferation. Knockout of Zeb1 reduces corneal inflammation and NV but may delay epithelial wound healing. In addition, ZEB1 may affect corneal scar formation after injury. On one hand, the expression of ZEB1 is under the control of different positive signaling pathways like TGF-β and negative regulators such as miRNAs. ZEB1 could also be an important target for many diseases, including cancer, fibrosis, and corneal injury repair. On the other hand, ZEB1 as a master regulator also regulates many signaling pathways, and the modulation of ZEB1 may affect cell and tissue normal biological functions, which may complicate the therapeutic utilization of ZEB1 inhibitors.

## Data Availability

Not applicable.
